# Biopolymeric Nanoparticles–Multifunctional Materials of the Future

**DOI:** 10.3390/polym14112287

**Published:** 2022-06-04

**Authors:** Andrey A. Vodyashkin, Parfait Kezimana, Alexandre A. Vetcher, Yaroslav M. Stanishevskiy

**Affiliations:** 1 Institute of Biochemical Technology and Nanotechnology, Peoples Friendship University of Russia (RUDN University), 6 Miklukho-Maklaya Str., 117198 Moscow, Russia; kezimana-p@rudn.ru (P.K.); stanyar@yandex.ru (Y.M.S.); 2 Department of Agrobiotechnology, Peoples Friendship University of Russia (RUDN University), 6 Miklukho-Maklaya Str., 117198 Moscow, Russia; 3 Complementary and Integrative Health Clinic of Dr. Shishonin, 5 Yasnogorskaya Str., 117588 Moscow, Russia

**Keywords:** biopolymeric nanoparticles, biodegradable, synthesis, applications, medicine, agriculture

## Abstract

Nanotechnology plays an important role in biological research, especially in the development of delivery systems with lower toxicity and greater efficiency. These include not only metallic nanoparticles, but also biopolymeric nanoparticles. Biopolymeric nanoparticles (BPNs) are mainly developed for their provision of several advantages, such as biocompatibility, biodegradability, and minimal toxicity, in addition to the general advantages of nanoparticles. Therefore, given that biopolymers are biodegradable, natural, and environmentally friendly, they have attracted great attention due to their multiple applications in biomedicine, such as drug delivery, antibacterial activity, etc. This review on biopolymeric nanoparticles highlights their various synthesis methods, such as the ionic gelation method, nanoprecipitation method, and microemulsion method. In addition, the review also covers the applications of biodegradable polymeric nanoparticles in different areas—especially in the pharmaceutical, biomedical, and agricultural domains. In conclusion, the present review highlights recent advances in the synthesis and applications of biopolymeric nanoparticles and presents both fundamental and applied aspects that can be used for further development in the field of biopolymeric nanoparticles.

## 1. Introduction

Nanoparticles are very promising materials with applications in various industries. Their active use is mainly due to their ultra-small size, easy functionalization, simple production methods, economical production, and easy scalability of their synthesis. They are used in industries such as the paper industry [1], water purification [2], catalysis [3], drug delivery [4], antibacterial products [5], and the food industry [6].

Materials with specific physical and chemical properties at the nano scale can potentially penetrate tissues, cells, and organelles, and interact with functional biomolecular structures, causing toxicity [7,8]. The toxicity of nanoparticles is affected by several parameters, such as their size, nature, surface functionalization, etc. [9,10].

Metal-based nanoparticles are the most popular nanoparticles found in commercially available products [11], but many of them are highly toxic to the human body [12,13,14], so more stringent quality and safety controls are needed for their use. Moreover, it is also necessary to strictly regulate the use of these particles in various applications [15,16]. In addition, metal nanoparticles can harm the environment (e.g., soil, water) and have a negative impact on the life of organisms therein [17,18]. It is worth mentioning that the use of metal nanoparticles for biomedical purposes is severely limited by the inability to excrete or metabolize them in the human body [19].

In this regard, biodegradable nanoparticles are needed not only for biomedical purposes, but also to improve the safety of various industries [20,21]. Biological polymeric compounds present several properties, such as biocompatibility, antioxidant, and antibacterial properties, photoprotection, and active surface functionality [22,23,24], making them good candidates for the development of nanomaterials with biological activities. 

Among the biodegradable nanoparticles, the most common are biopolymeric nanoparticles (BPNs), which are nanoparticles that are constructed from the natural polymers found in biological species such as proteins (e.g., collagen, gelatin, β-casein, zein, and albumin), protein-mimicking polypeptides (e.g., cationic polypeptides such as polylysine and polyornithine [25]), and polysaccharides (e.g., hyaluronic acid, chitosan, alginate, pullulan, starch, and heparin). These nanoparticles from various biopolymers are currently being created to solve problems related to toxicity, biocompatibility, and biodegradability. Biopolymeric nanoparticles (BPNs) are also applied in various industries, as are metallic NPs, but the former has increased safety and are environmentally friendly [26,27,28]. Currently, BPNs are actively used in the food industry, for biomedical purposes, in the development of diagnostic tools, and in the household chemical industry [29,30,31].

Although this manuscript concentrates on polysaccharide-based nanoparticles (e.g., chitosan nanoparticles, cellulose nanoparticles, etc.), we should also note that polypeptide-based nanoparticles have been reported as ideal materials for gene and drug delivery, due to their versatile traits, including excellent biocompatibility, biodegradability, low immunogenicity [32], precise secondary structure conformations, and self-assembling properties [33]. Moreover, with their bio-mimicked nature and surface functionalization, the use of polypeptide-based nanomaterials can help minimize adverse nano–bio interactions. For example, smooth excretion of PEG-polypeptides from the body via the biliary route within 3 h after their intended biomedical use further empowers their biocompatible nature [34]. 

The simplicity of carbohydrate-based BPNs’ methods of preparation, along with their wide application in various industries, has attracted several researchers, and there has been an increasing number of papers reporting on their synthesis, parameters, and applications. In this review, we discuss the main methods of their preparation, and their potential use in life sciences—from the delivery of biomolecules in organisms, diagnostic materials, and antibacterial agents, to improving agricultural production. Thus, this review presents a systematized analysis of the synthesis and application of BPNs.

## 2. Main Methods of Synthesis of Biodegradable Polymeric Nanoparticles

To date, several methods for the preparation of BPNs with different shapes and surface charges have been reported. The properties and, consequently, the application of BPNs depend on their shape, as spherical nanoparticles have been reported to have a high potential for cell penetration [35]. Therefore, there are requirements related to the shape of nanoparticles prepared from biodegradable polymers—especially those used as delivery agents in the human body. This requirement is of particular importance when choosing the preparation method and is discussed in the section on the synthesis of biodegradable polymeric nanoparticles.

### 2.1. The Ionic Gelation Method

**Figure 1:**
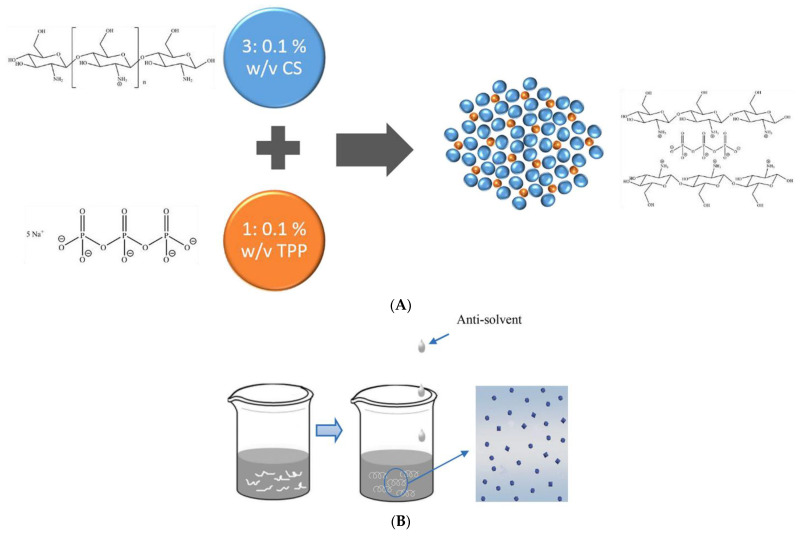
(**A**) Crosslinking of charged chitosan with sodium tripolyphosphate, reprinted with permission from [38], 2018, Elsevier. (**B**) Schematic illustration of the nanoprecipitation method, reprinted with permission from [44], 2019, Elsevier.

Gelation is the process of a polymer solution’s transition from liquid to solid form under the influence of polymer crosslinking reactions taking place in the solution. This process has been used since ancient times, and in recent years has been actively used to prepare BPNs [36]. Ionic gelation is a process that takes place under the influence of electrostatic interactions between ionic polymers and crosslinking agents, as illustrated in Figure 1. This method produces stable nanoparticles of chitosan, starch alginate, cellulose, and other biopolymers. Nanoparticles obtained via the ionic gelation method show good encapsulation efficiency and preservation of the bioactivity of the embedded molecules [37,38,39,40].

For the preparation of chitosan nanoparticles, Fan et al. demonstrated that the optimal ratio of chitosan to sodium tripolyphosphate is 3.3:1, the optimal pH of the solution is 4.7–4.8, and the optimal concentration of acetic acid solution is 0.2 mg/mL. These conditions make it possible to obtain nanoparticles of 138.1 nm in size, with the narrowest distribution [41].

Chitosan nanoparticles have also been obtained via ionic gelation, for which chitosan was dissolved in a 1% acetic acid solution to obtain a 0.1% solution (*w/v* and adjusted to a certain pH with a NaOH solution. A 0.1% solution of sodium tripolyphosphate in deionized water was prepared separately. While stirring, sodium tripolyphosphate solution was added to the chitosan solution at a rate of 0.1 mL/min, until a certain mass ratio of chitosan to sodium tripolyphosphate was reached. After adding the sodium tripolyphosphate solution, the resulting mixture was subjected to ultrasound for 10 min. It was found that factors such as the molecular weight of chitosan, the pH of the chitosan solution, and the mass ratio of chitosan to sodium tripolyphosphate affect the size of the resulting nanoparticles. The smallest particle size of 26 nm can be obtained by using low-molecular-weight chitosan, bringing the pH of the chitosan solution to 4.6 and the mass ratio of chitosan to sodium tripolyphosphate to 3:1. Moreover, the use of low-molecular-weight chitosan makes it possible to not use ultrasonic effects to obtain nanoparticles of a given size [42].

Anand et al. also obtained chitosan NPs via ionic gelation. In their method, they brought the pH of the chitosan solution to 6.0, and spherical nanoparticles with a wide distribution of 8–80 nm were obtained [43].

Ji et al. obtained chitosan nanoparticles with encapsulated amylase via ionic gelation [45]. A solution of carboxymethyl short-chain amylose was added to chitosan dissolved in an aqueous solution of acetic acid and stirred continuously for 6 h at room temperature. After that, the formed complex nanoparticles were isolated by centrifugation. The result was spherical nanoparticles with an average size of 262 nm. The work of Ji et al. demonstrates that the ionic gelation method allows loading of biodegradable polymer nanoparticles with active substances in the process of nanoparticle synthesis [45].

Anita et al. further demonstrated the possibility of preparing a complex of biodegradable polymer–metal nanoparticles and loading them with biomolecules using the ionic gelation method [46]. Copper nanoparticles were obtained by mixing copper sulfate, sodium hydroxide, and starch, which was used as a capping agent. To increase the functionality of the nanoparticles, microencapsulation was performed, which consisted of coating the nanoparticles with calcium alginate. This process was performed using the method of ionic gelation, the essence of which was to mix sodium alginate gel and copper nanoparticles, and to atomize calcium chloride into this system.

Qing et al. reported a study describing the preparation of negatively charged carboxymethylene branched starch nanoparticles via ionic gelation [37]. Starch and sodium hydroxide were added to ethanol, and the mixture was heated to 40 °C and incubated under stirring for one hour. Then, a mixture of monochloroacetic acid dissolved in ethanol was slowly added, after which the pH of the reaction mixture was adjusted to 12.0. The reaction mixture was incubated at 40 °C and under constant stirring for 4 h. At the end of the reaction, the pH of the mixture was adjusted to neutral with hydrochloric acid. The nanoparticles were isolated by centrifugation, and then washed with ethanol. As a result, starch nanoparticles with a spherical shape and a size of 50 to 100 nm were obtained.

Qing et al. also reported another way of preparing starch nanoparticles via ionic gelation, whereby a suspension of oxidized branched starch was gelatinized and slowly added to a solution containing calcium ions under stirring at room temperature, and then incubated for 2 h. The result was spherical starch nanoparticles with sizes ranging from 30 to 50 nm. It was noted that as the concentration of the calcium ion solution decreased, the size of the nanoparticles became smaller, opening the possibility of adjusting the size of the resulting particles [47].

The method of ionic gelation also makes it possible to obtain nanocomposites consisting of two biodegradable polymers. Subramanian et al. obtained composite nanoparticles consisting of chitosan and starch. Chitosan and starch were dissolved in an aqueous 2% acetic acid solution and, after complete dissolution, Tween-80 was added for uniform dispersion. A solution of a pre-synthesized anticancer agent and sodium tripolyphosphate was added to the mixture. The particles were isolated by centrifugation, and then lyophilized. As a result, nanoparticles of spherical shape, ranging in size from 173 to 297 nm, were obtained [48].

Nait Mohamed and Laraba-Djebari obtained alginate nanoparticles via ionic gelation [49]. Calcium chloride solution was slowly added to sodium alginate solution at room temperature and under constant stirring, and the mixture was incubated for one hour. The nanoparticles were isolated by centrifugation. As a result, nanoparticles of spherical shape, with a wide distribution of 150–350 nm, were obtained.

Stable nanoparticles of chitosan, alginate, starch, and other biopolymers can be obtained via the ionic gelation method. Nanoparticles obtained via this method show good encapsulation efficiency, small size, and high colloidal stability. Studies demonstrating the ability to adjust the size and distribution width of the resulting nanoparticles have been carried out.

### 2.2. The Nanoprecipitation Method

The nanoprecipitation method is a simple and fast process that differs from emulsion-based methods (e.g., emulsion–diffusion, emulsion–evaporation, and salting-out methods) in that no precursor emulsion is required. In practice, the hydrophobic solute (i.e., polymer or lipid molecules) is first dissolved in a polar organic solvent (usually ethanol, acetone, or THF). This solution is then added to a large amount of non-solvent (usually water) of the dissolved substance, with which the polar solvent is mixed in all ratios. The mixed binary solution becomes a non-solvent for the hydrophobic molecules, and the system evolves toward phase separation, resulting in the formation of hydrophobic solute particles (Figure 1B). The organic solvent can then be removed by evaporation. The formation of nanoparticles by nanoprecipitation ensures high drug content due to the coating of the nanoparticle surface with protective agents [44].

The resulting nanoparticles are separated by centrifugation or removal of the solvent with a rotary evaporator, and then washed with non-solvent (anti-solvent) or its mixture with distilled water, and subjected to lyophilic drying [50,51].

This method opens the possibility of obtaining nanodrug delivery agents. Moreover, instead of using multistep methods for obtaining nanoparticles and then incorporating the reagent into them, it is possible to introduce the drug into the nanoparticles directly during the nanoprecipitation process. El-Naggar et al. obtained starch nanoparticles with encapsulated diclofenac sodium via nanoprecipitation. Starch was dissolved in sodium hydroxide solution and incubated under stirring and at room temperature for 30 min, and then a mixture of diclofenac sodium and Tween-80 dissolved in distilled water was added to the resulting suspension. After 30 min, sodium tripolyphosphate solution was added, and the mixture was stirred continuously for 2 h. After 2 h, absolute ethanol was added to the mixture, causing the starch nanoparticles to precipitate. The formed nanoparticles were centrifuged and washed with a mixture of ethanol and water. As a result, spherical nanoparticles with an average diameter of 21.04 nm were obtained. The obtained nanoparticles were used as a delivery model for sodium diclofenac [52].

In the process of implementing the nanoprecipitation method, it is possible to regulate the size of the resulting nanoparticles. The solvent system used to dissolve the starch has a direct influence on the size of the resulting nanoparticles. Chin et al. obtained starch nanoparticles via nanoprecipitation. Starch was dissolved in a system of sodium hydroxide and urea solutions. The presence of sodium hydroxide in the solvent system is necessary for hydrolysis and for breaking the hydrogen bonds of the starch molecules. Urea plays an important role in preventing the self-association of starch molecules, leading to their increased solubility. The resulting mixture was slowly added to absolute ethanol and stirred at room temperature for 30 min. The resulting starch nanoparticles were isolated by centrifugation and washed with absolute ethanol to remove alkali and urea residues. The resulting nanoparticles were spherical in shape, ranging in size from 300 to 400 nm. The authors also demonstrated that adding the surfactant Tween-80 to the solvent system can reduce the size of the obtained particles to a range of 250–300 nm, and added hexadecyl(cetyl)trimethylammonium bromide to 150–200 nm [53].

Chin et al. explained the importance of the presence of a component such as urea in the starch solvent. However, it is worth highlighting a work that searched for the optimal concentrations of the components in the starch solvent. G. Gutierrez et al. obtained starch nanoparticles via nanoprecipitation. Their work studied the effects of the concentrations of the components of the starch solvent system, as well as the effect of the rate of addition of starch solution to absolute ethanol, on the size of the resulting particles. Starch was dissolved in an aqueous solution of sodium hydroxide and urea with different concentrations of components, and stirred at 80 °C for 30 min, after which the resulting mixture was slowly introduced into absolute ethanol. Spherical nanoparticles, 13.9 ± 9.03 nm in size, were obtained when concentrations of 8% (*w*/*v*) and 10% (*w*/*v*) urea were observed in the solvent system for dissolving the starch, and with an injection rate of the starch solution into absolute ethanol of 4 mL/h [54].

The nanoprecipitation method uses dimethyl sulfoxide (DMSO) as an alternative solvent for starch. DMSO is able to break the inter- and intramolecular hydrogen bonds of starch before replacing the hydroxy hydrogen bonds with DMSO–starch hydrogen bonds [55,56].

Pan et al. obtained starch nanoparticles via nanoprecipitation; in contrast to the previously listed works, the authors used DMSO as a starch solvent, and used ultrasound treatment to grind the resulting particles. Starch was added to DMSO and continuously stirred for 10 min, and then treated with ultrasound for 1 h. Then, deionized water was slowly added to the mixture while stirring, treated with ultrasound for another 1 h, and the obtained nanoparticles were isolated by centrifugation. In this work, the authors subjected the resulting nanoparticles to dialysis to remove impurities, and then lyophilized them. As a result, nanoparticles of spherical shape and a size of 68.63 ± 9.21 nm were obtained [55].

In addition to obtaining starch nanoparticles, the method of nanoprecipitation is also used for obtaining chitosan nanoparticles. Sotelo-Boyas et al. dissolved chitosan in acetic acid solution, and a mixture of lime and methanol was added to the solution while stirring. The formed nanoparticles were isolated by removing the solvent with a rotary evaporator. No additional purification was used for the obtained nanoparticles. As a result, the formed nanoparticles had a spherical shape and a size of 5.8 ± 1.6 nm. The obtained nanoparticles were used to encapsulate lime essential oil; as a result, the composite was studied for its antibacterial properties [57].

Chitosan-precipitated nanoparticles, as well as starch nanoparticles, have the potential to be drug carriers [58]. Thyme essential oils have antibacterial properties due to the volatile components they contain [59], but due to their volatile nature, thyme ester oils lose their antibacterial properties over time. The possibility of storing volatile components is opened by their encapsulation in nanocarriers. Sotelo-Boyas et al. used chitosan nanoparticles obtained via nanoprecipitation as nanocarriers for thyme essential oil components. The encapsulation process was performed directly during nanoparticle production. Chitosan dissolved in an aqueous solution of acetic acid was slowly added to a mixture of thyme essential oil and methanol under stirring. The resulting nanoparticles were isolated by removing the solvent using a rotary evaporator. As reported by the authors, 68% of the components of thyme essential oil were incorporated into the nanoparticles. As a result, the obtained particles had a spherical shape and an average size of 6.4 ± 0.5 nm [60].

Thus, the nanoprecipitation method opens the possibility of obtaining nanocarriers consisting of biodegradable polymers such as starch, chitosan, etc. There are possibilities for encapsulating drugs in nanoparticles directly during nanoparticle synthesis. The size of the resulting nanoparticles can be controlled by such parameters as the concentration of the reaction system components, the rate of adding “anti-solvent” to the polymer solution, and the use of ultrasound treatment.

### 2.3. The Microemulsion Method

Microemulsions are isotropic, thermodynamically stable, transparent (or translucent) colloidal dispersions consisting of at least three components: a non-polar phase, a polar phase, and a surfactant. The structural organization of microemulsions is usually of the oil-in-water (M/W) or water-in-oil (W/M) forms [61]. The microemulsion method is based on the addition of an aqueous polymer solution to an organic solvent including a surfactant during homogenization to form a finely dispersed water-in-oil (W/AM) microemulsion, in which nanoparticles are deposited [54]. The microemulsion method makes it possible to obtain nanoparticles of biodegradable polymers [62,63].

Trinh and Hung obtained starch nanoparticles via the microemulsion method. An alkaline starch solution was added to ethanol containing Tween-80 and soybean oil under stirring. The mixture was continuously stirred for 1 h. The formed nanoparticles were isolated by centrifugation and lyophilized. As a result, nanoparticles of spherical shape, with an average size of 70–200 nm, were obtained [64].

The microemulsion method makes it possible to obtain nanocomposite compounds such as two or more polymers crosslinked together, or metal nanoparticles encapsulated in polymers. Wang et al. obtained magnetic chitosan nanoparticles via the microemulsion method. Chitosan was dissolved in hydrochloric acid solution, and cyclohexane, hexanol-1, and divalent iron salt were added to the resulting solution while stirring. To the resulting mixture, TX-100 was slowly added until the mixture became transparent. After that, the reaction mixture was continuously stirred and heated at 60 °C for 2 h. Then, the particles were separated by centrifugation, washed with distilled water and ethanol, and lyophilized. As a result, chitosan nanoparticles with Fe_3_O_4_ nanoparticles encapsulated within them were obtained; the average diameter of the nanoparticles was 10–20 nm. The obtained nanoparticles were used as an adsorbent for albumin protein [65].

Dubey et al. obtained a chitosan–alginate nanocomposite via the microemulsion method. Chitosan was pre-dissolved in an aqueous solution of acetic acid and alginate in distilled water. Two prepared solutions and paraffin oil—which was used as an oil phase—were mixed in one flask, and then stirred continuously for 30 min, after which sodium tripolyphosphate and calcium salt solution were added. After that, they were stirred continuously at room temperature for another 3 h. The formed nanoparticles were isolated by centrifugation and washed with acetone. As a result, nanoparticles of spherical shape with an average size of 65 nm were obtained. The obtained chitosan–alginate nanocomposite was used as an adsorbent for mercury ions in aqueous solutions [66].

The use of ionic liquids in the microemulsion method allows the substitution of polar and non-polar phases and surfactants. The use of ionic liquids constitutes green chemistry because there is the possibility of their reuse, making them a profitable tool for use in reaction media. Ionic liquids have a melting point below 100 °C, which allows them to be used as solvents [67]. Zhou et al. obtained starch nanoparticles via microemulsion crosslinking using ionic liquid. Starch powder was added to l-octyl-3-methylimidazolium acetate (an ionic liquid replacing the aqueous phase) and stirred under heating for 2.5 h. Next, cyclohexane was added to the mixture, after which a mixture of the surfactant TX-100 and butanol-1 was added while stirring until the mixture became transparent. Epichlorohydrin was added to the resulting mixture as a crosslinking agent. The mixture was then stirred at 50 °C for 3 h. After 3 h, the mixture was cooled to room temperature and the starch nanoparticles were precipitated with anhydrous ethanol under vigorous stirring, followed by centrifugation. The precipitate was washed thoroughly with absolute ethanol to remove unreacted epichlorohydrin, butanol-1, and TX-100. After that, the solid was centrifuged and dried in vacuo at 45 °C for 24 h. As a result, starch nanoparticles of spherical and oval shape, with an average size of 96.9 nm, were obtained [68].

Wang et al. obtained starch nanoparticles via microemulsion crosslinking, the technique of which was similar to that of Zhou et al., with the only difference being that the authors used 1-hexadecyl-3-methylimidazolium bromide as the ionic liquid instead of l-octyl-3-methylimidazolium acetate. As a result, nanoparticles of indeterminate shape, with an average diameter of 80.5 nm, were obtained [69]. The use of ionic liquids makes it possible to obtain nanoparticles of biodegradable polymers up to 100 nm in size.

The microemulsion method is complex in terms of its implementation since the process is implemented in several steps. This method is suitable for obtaining nanodrugs based on biodegradable polymers. The use of ionic liquids in this method opens opportunities for technical simplification and cheaper process.

### 2.4. The Coacervation Method

Coacervation is the spontaneous formation of a superdense liquid phase from a homogeneous macromolecular solution with low solvent affinity. During coacervation, a homogeneous solution of charged macromolecules undergoes liquid–liquid phase separation, resulting in a polymer-rich dense phase coexisting with the supernatant. The two liquid phases do not mix, but strongly interact, allowing the formation of nanoparticles of different biopolymers [70]. 

Starch nanoparticles have been prepared via coacervation, with solutions of pre-modified starch of two kinds—positively charged (with an amino group) and negatively charged (with a carboxyl group)—being mixed at different molar ratios under stirring and at room temperature. Nanoparticles were formed within 15 min. The formed nanoparticles were centrifuged and lyophilized. With this method, spherical nanoparticles were obtained, and the optimal ratio for obtaining nanoparticles with the smallest size of 138.9 ± 0.3 nm was found to be 1:3 [71]. Studies have shown that with a similar methodology, composite nanoparticles of biodegradable polymers of chitosan and carboxymethyl starch can also be prepared [72].

Another method of coacervation is the saturation of the polymer solution followed by the formation of the coacervate phase. Tavares et al. obtained chitosan nanoparticles via the simple addition of sodium sulfate solution to a chitosan solution with fixed volumes and concentrations under magnetic stirring [73].

Patra et al. used a modified coacervation method, using a binary mixture of acetone and ethanol to initiate phase separation. This approach allowed the formation of a compact structure of the polymer chain, which contributed to the reduction in the particle size. Gelatin nanoparticles were obtained via the binary coacervation method. A mixture of acetone and alcohol was added to an aqueous gelatin solution heated to 40 °C, observing a 1:1:1 volume ratio of the components. Next, an ethanol solution of the crosslinking agent—glutaric aldehyde—was added to the mixture. Moreover, the aqueous gelatin solution was brought to different pH values—ranging from 3 to 11—to study the effect of pH on the size of the resulting particles. The resulting particles were washed several times with distilled water, treating the system with ultrasound for 5 min during each wash cycle, and then centrifuged at 2000 rpm. As a result, the minimum particle size was achieved at pH = 7, with a size of 55.67 ± 43.74 nm [74]. It is worth noting that this method can produce nanoparticles of biopolymers with a very small size, but a wide distribution, which may limit some of their applications.

### 2.5. The Electrospray Method

**Figure 2:**
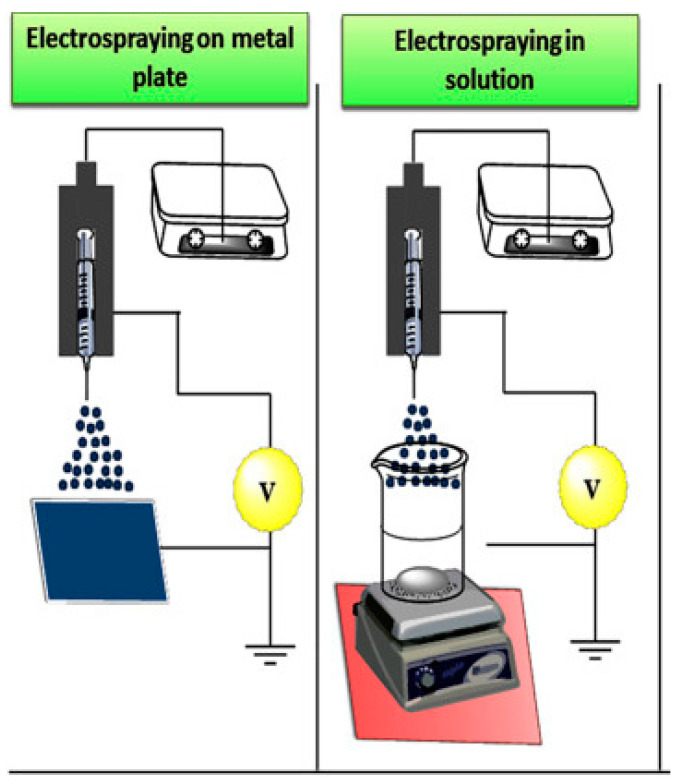
Illustration of a setup of the electrospray method, reprinted with permission from [75], 2018, Elsevier.

The electrospray method is based on forces such as electromechanical and hydrodynamic forces. The basic elements of this method are a syringe pump, a nozzle equipped with a cooling jacket, a high-voltage power supply, and a metal manifold. Important factors in this process are the distance from the needle to the manifold, the flow rate through the nozzle, and the applied voltage. In this method, a solution of the substance from which the nanomaterial is to be produced flows through the nozzle under voltage and is sprayed onto the collector at varying speeds. The collector, in turn, acts as a grounding agent. Special drums or solutions can also be used instead of the collector. This method is traditionally used to create drug nanocrystals (Figure 2) [75], but there has also been works on obtaining nanoparticles of biodegradable polymers via this method.

Applying an electric charge to the polymer solution plays an important role in electrospraying, and the use of a high electric voltage deforms the droplet interface and results in nanometer-sized droplets [76]. The proposed mechanism is that when an electric field is applied to a liquid droplet, an electrostatic force in the droplet—called the Coulomb force—is generated, which competes with the adhesion force in the droplet. When the applied Coulomb force overcomes the adhesion force of the droplet, the surface tension is released, and nanodroplet formation occurs [77].

Wang et al. obtained chitosan nanoparticles encapsulating tea polyphenols. A solution of chitosan in acetic acid was prepared, and then tea polyphenols were added at various mass ratios, and the pH of the mixture was maintained in the range of 4.6–4.7. A solution of sodium tripolyphosphate was prepared separately, to which the mixture of chitosan and tea polyphenols was fed through a nozzle with a diameter of 0.5 mm; the distance from the nozzle was 5 to 15 cm, the feed rate of the mixture was 0.25–1 mL/h, and the voltage applied to the nozzle was 10–20 kV. The formed nanoparticles were isolated by centrifugation and lyophilized. As a result, the smallest size of chitosan nanoparticles with encapsulated tea polyphenols was 128 ± 13 nm, using the following parameters: nozzle-to-solution distance of 5 cm, spray rate of 1 mL/h, and voltage of 15 kV [78].

Chitosan nanoparticles were also obtained by electrospraying using a special unit for voltage generation, with a syringe with an internal needle diameter of 0.65 mm and a working distance of 10 cm. Chitosan dissolved in acetic acid was fed at a rate of 0.2 mL/h to 1.1 mL/h. After that, it was dried in a vacuum. It is worth noting that solutions of different concentrations and molecular weights can be used to produce chitosan nanoparticles [79].

## 3. Main Applications of Biodegradable Polymeric Nanoparticles

Based on their diverse range of molecular and physicochemical properties, biopolymeric nanoparticles exhibit a broad range of functional attributes. They are therefore used in different areas, including medical, agricultural, and industrial applications. In the present review, we concentrate on the medical and agricultural uses. 

### 3.1. Drug Delivery Using Nanoparticles of Biodegradable Polymers

Nanoparticles of biodegradable polymers are unique systems for the delivery of various drugs into the human body [80,81]. The small size and easy possibility of functionalization of nanoparticles allow them to be used as a universal system for the delivery of various drugs [82,83]. Nanoparticles of polymers can be loaded with a high concentration of a drug substance, but due to its inclusion in the nanoparticles it does not cause harm to the body [84,85]. The drug included in nanoparticles is protected from the body’s environment, helping it to maintain its bioactivity [86,87,88].

Polysaccharide-based nanoparticles can reduce uptake by the mononuclear phagocyte system compared to other types of NPs, prolonging the in vivo NP residence time and increasing the likelihood of disease focus accumulation [89]. Polymer nanoparticles allow increased accessibility, as well as increased hydrophilicity of the therapeutic agent [90]. Moreover, by incorporating various drugs into polymer nanoparticles, targeted delivery systems can be created, allowing the drug to be delivered to the location of the tumor or thrombus and, thus, greatly increasing the therapeutic effect [91,92]. 

One example of biopolymer nanoparticles used for drug delivery is starch nanoparticles. Xie et al. prepared cassava starch nanoparticles via the microemulsion method, using POCl_3_ as a crosslinking agent. In this work, the adsorption of the anticancer drug paclitaxel (PTX, C_47_H_51_NO_14_) onto starch nanoparticles was studied, and the kinetics of the process were studied; 94.19% of free PTX was released from the dialysis bag in 0.9% saline (pH = 7) within 8 h. Compared with free PTX, the release rate of CA-CSNP-loaded PTX was 17.04% after 8 h and 37.61% after 96 h under the same conditions, showing the possibility of prolonging the therapeutic effect of the drug, which can improve the effectiveness of antitumor therapy [93]. All of this confirms the high prospects for the use of starch nanoparticles as drug carriers to provide a targeted effect on tumor cells.

Alp et al. proposed a method of producing starch nanoparticles synthesized using the emulsion–solvent diffusion method, used to deliver CG-1521 (7-phenyl-2,4,6-heptatrienoylhydroxamic acid), which is a histone deacetylase inhibitor (HDAC). The authors demonstrated the release kinetics of the drug, consisting of a release of approximately 40% within the first 10 h and 64% within 120 h. The authors demonstrated localization of NPs in the cytoplasm and perinuclear region of MCF-7 cells, indicating that they were effectively absorbed by the cells after 12 h. During the study, it was found that the cytotoxic activity of CG-1521 incorporated into starch nanoparticles was more than three times higher compared to the pure CG-1521 preparation [94]. This system demonstrated high cytotoxic activity against MCF-7 cells. In addition, it was shown that it is possible to create systems with prolonged action, which can increase the effectiveness of the therapy.

Another promising material for drug delivery is chitosan nanoparticles; de Oliveira Pedro et al. developed a method for producing chitosan nanoparticles based on the self-assembly process, with quercetin incorporated into them. Due to the non-polar molecules in the core of chitosan nanoparticles, it is possible to include hydrophobic molecules such as quercetin. The authors studied the kinetics of quercetin’s release, and suggested mechanisms that affect the release of this drug. Chitosan nanoparticles with quercetin demonstrated an inhibitory effect against MCF-7 cells comparable to that of free quercetin. With a confocal laser microscopy, it was found that some of the nanoparticles accumulate on the cell membrane, while others are internalized. In addition, these chitosan nanoparticles were shown to have suitable compatibility with blood which, together with their biodegradability, allows them to be used as an effective delivery system for hydrophobic therapeutic molecules [95].

Theoretical calculations of real systems can help to create more efficient drug delivery systems, as well as increasing the therapeutic effects of existing drugs. Rahbar et al. performed quantum chemical calculations on the use of chitosan nanoparticles as carriers for the anticancer drug 5-fluorouracil. Negative values of the bonding energies showed that the functionalization of CΓT PFs is energetically advantageous, and the solvation energies showed an increase in the solubility of 5-fluorouracil. Such a system would help increase the solubility of the hydrophobic anticancer drug, as well as reducing the burden on the whole body by incorporating 5-fluorouracil into chitosan nanoparticles [96].

**Figure 3:**
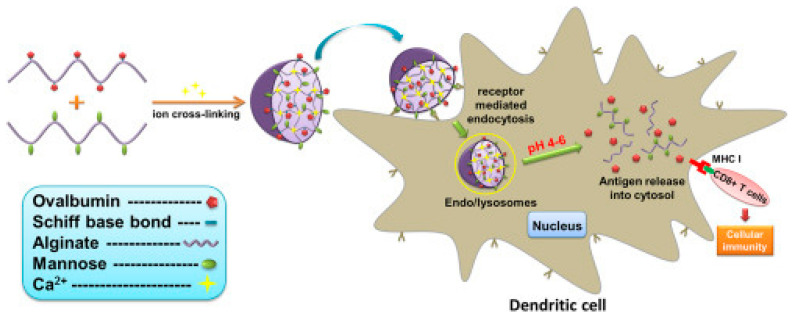
Illustration of the potential use of functionalized alginate nanoparticles as a nanovaccine for cancer immunotherapy. Reproduced with permission from [97], 2017, Elsevier.

Zhang et al. proposed functionalized alginate nanoparticles crosslinked with calcium ions and modified with mannose to deliver ovalbumin. Ovalbumin was attached via a pH-sensitive bond and used as a model antigen (Figure 3). The release of ovalbumin from the alginate nanoparticles was in the region of 30% after 43 h, at a pH of 5.5. Using confocal microscopy, alginate NFs were found to facilitate antigen transport, uptake, and delivery to the cytosol of dendritic cells. In vivo experiments on laboratory mice proved that alginate encapsulated in nanoparticles can be gradually transported from the injection site to the draining lymph nodes, and can remain there for several days, providing a targeted and prolonged therapeutic effect. The antitumor effect was evaluated in OVA-expressing E.G7-OVA cells of mice. The average tumor weight was at least four times less than in the control group and the group with free ovalbumin, showing the synergistic effect of the inclusion of ovalbumin in nanoparticles of modified alginate. This system allows a significant increase in antigen absorption, provides increased efficacy of antitumor therapy, and can also provide a prolonged therapeutic effect (Figure 3) [97]. 

Cellulose nanoparticles can also successfully serve for drug delivery to the human body. Suk Fun Chin et al. demonstrated a simple method for producing cellulose nanoparticles and optimized the loading efficiency and studied the release kinetics of the substance with a model hydrophilic drug [98].

**Figure 4:**
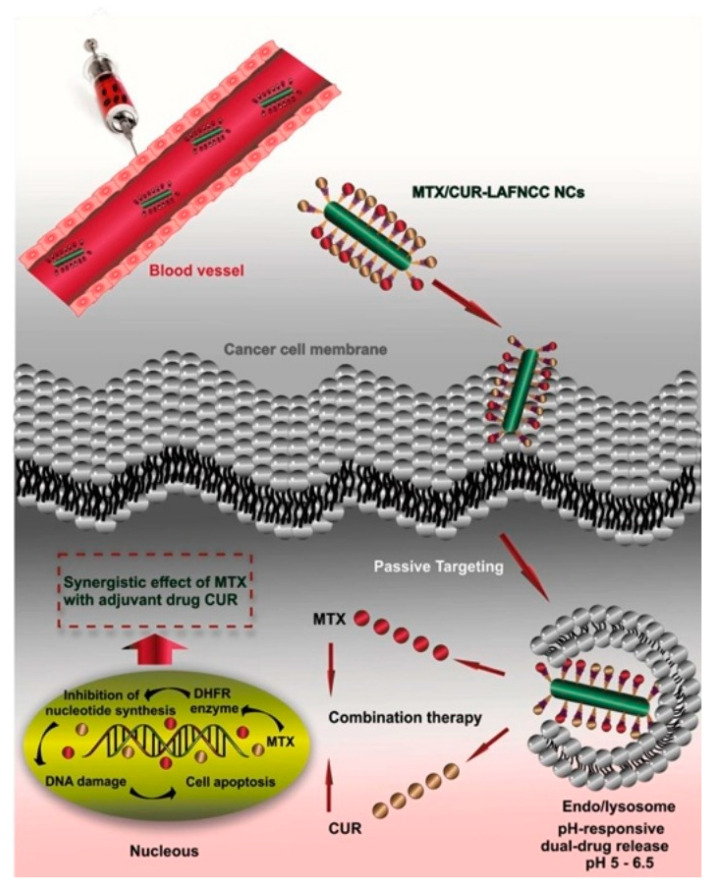
Schematic diagram for dual-drug delivery of a lysine-embedded cellulose-based nanosystems. Reproduced with permission from [99], 2020, Elsevier.

Modified biopolymers are increasingly being used in the delivery of various therapeutic agents into the body. Various theranostic and therapeutic systems are particularly promising. An example of such a system could be amino-functionalized nanoparticles of nanocrystalline cellulose modified with 1-lysine [99]. In their work, Moghaddam et al. presented a method of obtaining nanocrystalline cellulose functionalized with aminoprapyl-3-methoxysilane and conjugated with 1-lysine. This nanocarrier was used for simultaneous delivery of two drugs: methotrexate (MTX) and curcumin (CUR). The average size of the nanoparticles was 240 nm, as confirmed by data from DLS and SEM. The critical parameter for any injectable drug is the hemocompatibility of the carrier, which allows its use in the form of injections. Modified cellulose nanoparticles had absolute safety when injected at a concentration of 1000 µg/mL. The cellulose nanoparticles showed high cytotoxicity against MCF-7 andMDA-MB-231 cells, with an IC_50_ of 2.253 and 15.34, respectively. It is especially noteworthy that the modified cellulose nanoparticles have a significant effect on cell morphology and cell number and exceed these figures for free drugs (according to a DAPI staining study) (Figure 4) [99]. Nanoparticles proved to be a highly effective platform for drug delivery via the bloodstream to fight cancer. In addition, the authors studied the safety and kinetics of drug release. This study showed the high promise of cellulose nanoparticles for use in the delivery of cytotoxic and antitumor drugs. 

Composite nanoparticles of biopolymers can also be actively used for the delivery of various drugs. Sorasitthivanukarn et al. demonstrated the possibility of creating alginate/chitosan nanoparticles obtained via ionotropic gelation and emulsification; three-level Box–Behnken statistical design was used to optimize the synthesis and loading parameters of curcumin di-glutaric acid as an antitumor agent. During exposure of curcumin di-glutaric acid immobilized in nanoparticles and in free form, it was proven that the nanocomposite material protects the antitumor agent from photodegradation. In an in vitro simulated experiment, it was found that the chitosan/alginate nanoparticles are not subject to degradation under conditions close to the gastrointestinal tract (data confirmed using DLS and SEM). In addition, these nanoparticles showed the ability to penetrate Caco-2 cells (used to evaluate the absorption of orally administered drugs across the gastrointestinal barrier) via the endocytic route. The chitosan/alginate nanoparticles showed a complete absence of cell toxicity, whereas the nanoparticles loaded with the model drug (curcumin di-glutaric acid) showed high cytotoxicity toward MDA-MB-23, HepG2, and Caco-2 cells [100]. 

The specifics of the chitosan/alginate nanosystems allow us to speak about their high potential for use as agents for the oral delivery of biologically active substances. This system shows absolute absence of toxicity to cells and high permeability inside GIT cells. Furthermore, nanoparticles can provide increased preservation of the therapeutic drug, as well as prolonged action of the drug substance.

Alkholief M. proposed a method to create self-assembled lecithin/chitosan nanoparticles used for the delivery of various therapeutic drugs. The authors developed and optimized the nanoparticle parameters using Box–Behnken design. The chitosan/lecithin nanoparticles had the ability to exert prolonged action through gradual release of the drug. This system, based on mathematical modeling, showed promise in the delivery and prolongation of drug action, but further in vivo experiments are needed to confirm the effectiveness of this system on living subjects, and a hemocompatibility study is needed to form a complete picture of the possibility of using lecithin/chitosan nanoparticles on living subjects [101].

Biopolymer nanoparticles can be used to deliver a range of therapeutic agents into the human body. Biopolymer nanoparticles can protect the drug substance from the body’s environment, as well as providing prolonged release of the drug, which can increase the effectiveness of the therapy many times over; in addition, biopolymer nanoparticles can be used not only for venous delivery, but also for oral and ophthalmic delivery of drugs [102,103]. In combination with their nano scale and biocompatibility, biopolymer nanoparticles can play a leading role in the delivery of drugs to the human body.

### 3.2. Diagnostic Systems Based on Nanoparticles of Biodegradable Polymers

Systems based on biodegradable polysaccharides have been successfully used as the basis for various diagnostic and theranostic systems. In recent years, the use of polysaccharide nanoparticles for the diagnosis of various diseases has become relevant [104,105]. Nanoparticles of biopolymers can be loaded with various substances, providing highly synergistic effects and diagnostic/therapeutic efficiency [106,107]. 

Nanoparticles of chitosan and its various modifications can successfully serve for theranostics of hepatocellular carcinoma. Chitosan nanoparticles can control drug release and increase drug stability. Additionally, due to the positive charge of the amino group, they can better bind to the negatively charged membranes of tumor cells, increasing the theranostic effect [108].

**Figure 5:**
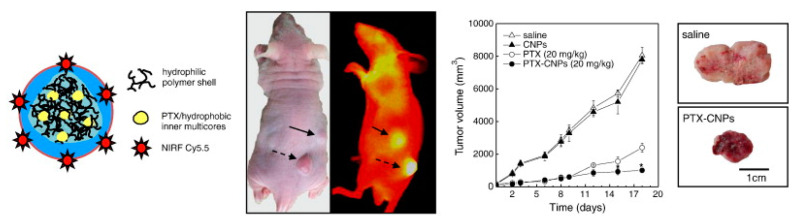
Schematic diagram of chitosan-based nanoparticles used in cancer diagnosis and therapy. Reproduced with permission from [109], 2010, Elsevier.

Kim et al. proposed a way to produce chitosan nanoparticles modified with Cy5.5 (IR dye) and encapsulating paclitaxel (an anticancer drug). Tumor-binding specificity was tested in vivo on squamous-cell carcinoma cells of SCC7 mice. One day after the injection of modified chitosan nanoparticles (CNPs), early-stage tumors were clearly separated from the surrounding normal tissue, demonstrating CNPs’ specificity with respect to tumor targeting, with the NIRF signal being proportional to the tumor size. Chitosan nanoparticles accumulated throughout the tumor tissue, which could be used for improved detection of tumor localization. When studying biodistribution, it was found that the NIRF signal per gram of each organ in tumor tissues was 4–7 times higher than in other organs. In vivo experiments on mice showed a high survival rate of mice that received paclitaxel incorporated into chitosan nanoparticles, as opposed to free paclitaxel (Figure 5) [109]. All of the above shows the possibility of using a system based on chitosan nanoparticles and paclitaxel as an effective theranostic system to diagnose cancer at early stages, deliver the drug, and monitor tumor parameters during treatment.

Nanoparticles (nanocrystals) of cellulose can also be used to create various diagnostic systems. Mahmoud et al. proposed a method for obtaining nanocrystalline cellulose derived from linen fibers, which had an average diameter of about 20 nm and a length of 120 nm. The authors studied the cellular uptake of cellulose nanoparticles incorporating the fluorescent dye rhodamine B isothiocyanate in HEK 293 and Sf9 cells. The nanoparticles were mainly dispersed throughout the cytoplasm, although some of them were identified by their vesicular structure. In the nuclei of both cell lines there was no significant amount of nanoparticles, indicating less interaction between these nanocrystals and the nuclear membrane. In addition, an MTT test was performed to determine the cytotoxicity of the nanoparticles with respect to the cells, and it was found that at a concentration of 100 µg/mL, the cell viability was 90% of that of the control sample. Nanocrystalline cellulose can be considered as a new generation of probes for bioimaging, given its large surface area of 150 m^2^/g and the ability to adjust the surface charge to penetrate the cell without causing cell damage [110].

Photoacoustic imaging (PAI)—a biomedical analysis technique that provides actionable information about the molecular characteristics of tissue—is a recently developed hybrid biomedical imaging technique for monitoring tumor angiogenesis and detecting skin melanoma, as well as for monitoring and diagnosing various other neoplasms [111]. 

**Figure 6:**
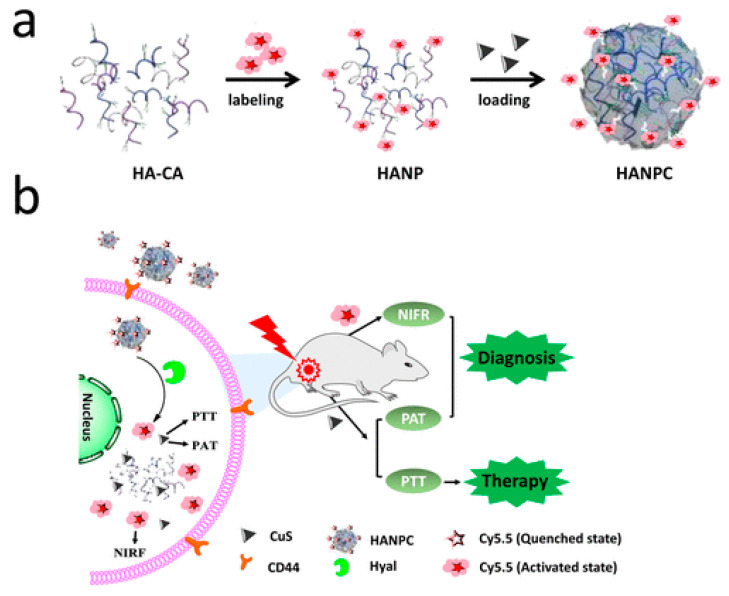
Illustration of (**a**) synthesis of activatable Cy5.5–HANP/CuS (HANPC) nanocomposites, and (**b**) in vivo applications. Reproduced with permission from [112], 2014, American Chemical Society.

Hyaluronic acid nanoparticles modified with Cy5.5 IR dye and copper (II) sulfide were used as a contrast agent for photoacoustic and fluorescent imaging. Hyaluronic acid nanoparticles were injected intravenously, and fluorescence images were obtained at various time points after injection. After the injection of hyaluronic acid nanoparticles with Cy5.5 and copper (II) sulfide, hyaluronic acid was cleaved by hyaluronidase in the tumor area, and the quenching effect between CuS and Cy5.5 was reduced, resulting in the recovery of the fluorescence signal over time (Figure 6) [112]. Photoacoustic imaging allows detailed examination of tumor blood vessels and increases the effectiveness of therapy. PA contrast at the tumor site increased over time and reached a maximum after 6 h; it is also worth noting that using only copper sulfide did not significantly increase the PA signal. When studying the biodistribution, in addition to the tumor, the authors detected a signal in the kidneys, which was associated with the biodegradation of hyaluronic acid nanoparticles. The main mechanisms to which the high selectivity towards tumor cells can be attributed are the effects of increased permeability and retention, as well as receptor-mediated endocytosis. Hyaluronidase overexpression in tumor cells can effectively degrade hyaluronic acid nanoparticles and, consequently, enhance strong fluorescence signals for detecting tumor localization. In addition, hyaluronic acid nanoparticles have shown promise as carriers of signal-enhancing substances for PAI [112].

### 3.3. Therapeutic and Tissue Engineering Applications

**Figure 7:**
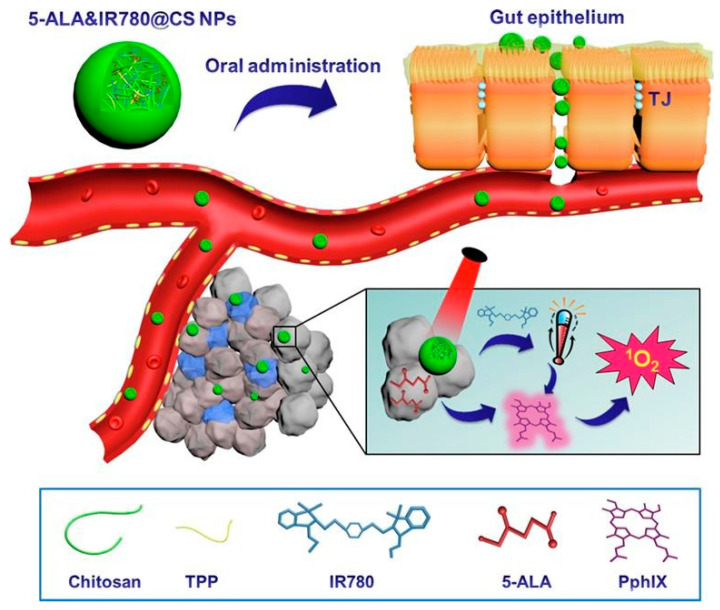
Schematic diagram of chitosan-nanoparticle-based 5-ALA and IR780 delivery for oral PTT and PDT in cancer treatment. Reproduced with permission from [113], 2020, Elsevier.

Chen et al. proposed a method to produce chitosan nanoparticles obtained via ionic crosslinking, which can be used for photothermal therapy. Then, the chitosan nanoparticles were modified with 5-aminolevulinic acid (5-ALxAl) and IR-780 iodide. When heating the modified nanoparticles in solution for 2 min, a temperature of 67.2 °C was recorded, which is a sufficient value for the application of these nanoparticles as an agent for photothermal (PTT) and photodynamic therapy (PDT). The authors performed a comparative analysis of the cytotoxicity of nanoparticles loaded alternately with 5-aminolevulinic acid (5-ALA) and IR-780 iodide and mixed with chitosan nanoparticles (Figure 7). Nanoparticles loaded with both drugs showed higher efficacy relative to other chitosan systems. Moreover, nanoparticles modified with two drugs showed greater areas of tumor necrosis, fragmentation, and tumor tissue damage compared to chitosan nanoparticles with the inclusion of one drug each. The biodistribution of chitosan nanoparticles and their safety for the human body were also studied in this work [113]. This work successfully demonstrated the high efficacy of chitosan nanoparticles in photothermal and photodynamic therapies and laid the foundation for the transition to medical practice with the use of nano-objects, due to the safety of this complex preparation of chitosan nanoparticles for anticancer therapy.

**Figure 8:**
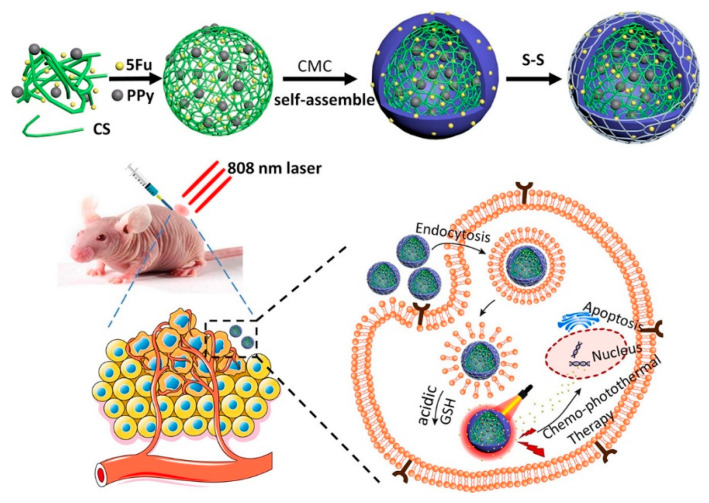
Schematic diagram of the synthesis of a drug-loaded complex of chitosan NPs and carboxymethyl cellulose, and its application in photothermal chemotherapy for tumor cells. Reproduced with permission from [114], 2022, Elsevier.

Biopolymeric nanoparticle composite materials can also be used for the treatment of tumors, and as agents in photothermal therapy. Wang et al. proposed a nanopolysaccharide composite consisting of chitosan nanoparticles loaded with polypyrrole and 5-fluorouracil nanoparticles, with an outer shell of carboxymethyl cellulose crosslinked with disulfide. The composite material had an average diameter of 254 nm. Using a laser of 808 nm and a power of 1.5 W/cm^2^, the temperature of the nanoparticle solution increased from 20 to 42 °C in 300 s, while the buffer temperature increased by 2 °C. In addition, the high photothermal stability and reproducibility of the effect caused by the nanopolysaccharide complex were proved (Figure 8). The photothermal conversion efficiency was calculated from the descending part of the curve and was 21.6. The nanopolysaccharide complex also exhibited antitumor properties, as confirmed by in vivo experiments [114].

Biopolymer nanoparticles can be successfully used as a basis for drug delivery in photothermal therapy, as well as having antitumor effects.

In recent years, the development of biocompatible and non-toxic nanoparticles that can be used in bone engineering, implants, and other biomedical systems has been relevant.

Chitosan induces mineralization during the differentiation of mesenchymal stem cells’ osteoblasts by enhancing genes related to mineralization and calcium binding, such as collagen type I alpha I, integrin-binding sialoprotein, osteopontin, osteonectin, and osteocalcin [115]. Chitosan nanoparticles, in contrast to chitosan, can also be used for the delivery of various drugs, as well as to provide stimulation of the immune system and antibacterial activity in this system. Jafary and Vaezifar proposed the use of chitosan nanoparticles obtained via ionic gelation to incorporate alkaline phosphatase enzymes. Overall, 71% of the alkaline phosphatase retained its native activity, with 55% protein–chitosan nanoparticle binding. The expression of the major bone markers osteocalcin, alkaline phosphatase, and collagen at day 7 increased 35-, 6-, and 13-fold, respectively, in the group with the addition of chitosan nanoparticles relative to the control group. At the same time, the formation of bone nodules and an increase in the size of the nodules during the differentiation of osteoblasts in the presence of immobilized ALP/chitosan nanoparticles were observed by days 7 and 14 of differentiation, as established by means of von Koss staining. At the same time, the number of bone nodules was many times higher than in untreated cells. This work proved that chitosan nanoparticles can be used as an effective component in bone tissue therapy. Simultaneous use with alkaline transferase increases the expression of genes that are associated with the mineralization and diffusion of osteoblasts [116]. 

Herdocia-Lluberes et al. proposed a method of creating a composite material based on nanohydroxyapatite and nanocellulose using the gelation method. The average size of these nanoparticles was 300 nm. The toxicity of these nano-objects at a concentration of 5 mg/mL was studied in relation to osteoblast cells. It was shown that nanocellulose/nanohydroxyapatite nanoparticles modified with the BMP-2 protein induced the increased growth of osteoblasts compared to the control group. This study demonstrated the possibility of using cellulose nanocomposites for bone tissue engineering [117].

**Figure 9:**
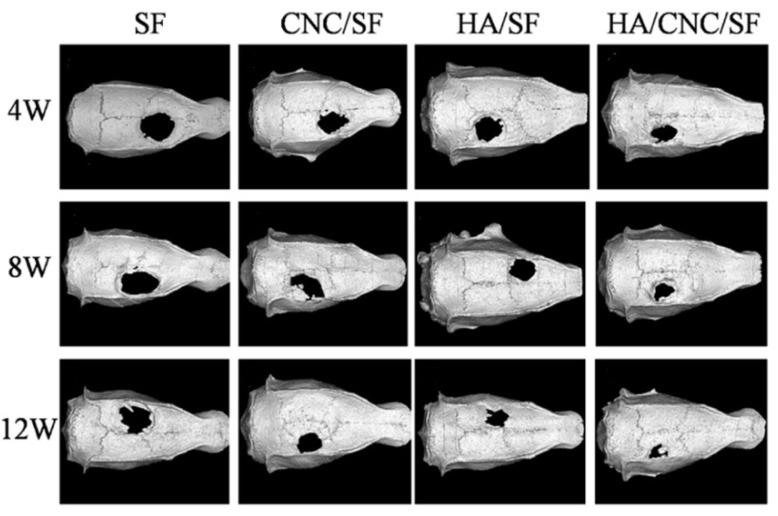
Illustration of the different repair results in rat calvarial defects assessed by micro-CT. Reproduced with permission from [118], 2011, Royal Society of Chemistry.

A similar system based on nanocellulose, hydroxyapatite, and silk fibroin has been proposed for the regeneration of a rat brain/skull defect (Figure 9) [118]. When investigating the effect of the nanocomposite on MC3T3-F1 cells’ viability, it was found that the cell viability at 5 and 7 days was much higher than that of samples without the composite material. The hybrid materials increased alkaline phosphatase activity at days 7 and 14 of the cell studies. In this work, new murine skull-bone formation was measured after injury using micro-CT. Rat skulls treated with the composite material showed significant growth at 12 weeks, relative to silk fibroin, as well as to compositions of silk fibroin–nanocrystalline cellulose and silk fibroin–nanohydroxyapatite. With the help of histological analysis, it was proven that in the formation of the skull modified with the composite material based on nanocellulose, hydroxyapatite, and silk fibroin, the main role is played by the large amount of new bone. High biocompatibility and efficacy in the repair of rat skull vault defects confirmed the prospects of composite materials based on nanocrystalline cellulose in bone tissue engineering.

Nanocellulose has also been reported to improve the functional properties of chitosan-based nanocomposites, since chitosan has poor mechanical properties that limit its applications in bone tissue engineering [119]. Nanoparticles are usually used in tissue engineering, and they are able to mimic the natural extracellular matrix, thus increasing the possibility of controlling scaffold features, such as biomechanical and biological properties [120,121].

### 3.4. Antibacterial Properties of Nanoparticles of Biodegradable Polymers

Antibacterial properties against various bacteria are necessary for use in various areas of human life. The antibacterial properties of various metal nanoparticles are widely known [122,123,124]. Metal nanoparticles are highly toxic to human cells, and can be harmful not only to bacteria, but also to humans [125,126]. Biocompatible antibacterial systems have high potential for current industrial applications. Biopolymer nanoparticles can exhibit antibacterial activity against several bacteria [127,128]. The mechanisms of action and toxicity against different strains manifest individually for each nanoparticle. One of the biopolymers exhibiting antibacterial activity is chitosan. The antibacterial properties of chitosan depend on the transfer of amino groups’ protons, which can react with the negative charge of the cell surface, leading to the destruction of the bacterial cell. Chitosan interacts with the bacterial membrane, changing the cell permeability [129,130].

Some authors have demonstrated that low-molecular-weight chitosan nanoparticles can penetrate the cell, bind to DNA, and inhibit the replication mechanism [131]. Chitosan nanoparticles exhibited higher antimicrobial activity than chitosan against a wide range of pathogens, including fungi as well as Gram-positive and Gram-negative bacteria. Chitosan nanoparticles obtained via ionic gelation were medium-sized and exhibited antibacterial activity against *N. gonorrhea*. Their MIC_50_ was 0.16 mg/mL at pH 5.5. Bacterial adhesion studies showed that chitosan nanoparticles reduced the adhesion of multidrug-resistant *N. gonorrhea* (WHO Q) to HELA cells at the lowest concentration of chitosan nanoparticles, and inhibited bacterial growth compared to cells not exposed to chitosan nanoparticles [132]. It is worth noting that when the pH was increased to 7.5, chitosan nanoparticle activity decreased, consistent with other reports in the literature [133]. Chitosan nanoparticles showed promise in their use as an antivaccine agent and were also cytocompatible and reduced bacterial cell adhesion.

The antibacterial properties of chitosan nanoparticles are widely used in industry. Mousavi et al. proposed a fabric conditioner using chitosan nanoparticles. This conditioner exhibited antibacterial properties against bacteria such as *Streptococcus mutans*, *Enterococcus faecalis*, and *Pseudomonas aeruginosa*, as well as the fungus *Candida albicans*. Complete inhibition of microbial growth was performed at a concentration of 2.5–10% of chitosan nanoparticles in the system [134].

Furthermore, various nanosystems based on chitosan in combination with various biopolymers have been used as a basis for antibacterial systems used in various industries [135,136].

### 3.5. Biodegradable Polymer Nanoparticles in Agriculture

The importance of nanotechnology has increased in all branches of science, including agriculture. With the goal of enhancing crop yield and providing food security, several researchers have turned to agricultural nanotechnology [137]. Studies have shown that the use of nanomaterials in agriculture can be oriented towards (a) plant nutrition by controlling the release of agrochemicals; (b) plant protection, e.g., release control of pesticides, detection of plant diseases; and (c) crop improvement, e.g., delivery of genetic materials [138]. Despite the advantages of nanomaterials, one of the parameters that usually hinders their use in agriculture is their toxicity, which has made the use of non-toxic, biocompatible, and biodegradable polymeric nanoparticles more advisable [137,138]. Therefore, several biopolymers—such as carbohydrates (e.g., chitosan, cellulose, starch)—have been used to prepare biopolymeric nanoparticles. In addition to the usual advantages of nanoparticles in agriculture, biopolymeric nanoparticles also play an important role in agriculture, as they have plant growth and plant protection properties [139]. Moreover, with their unique properties, biopolymeric nanoparticles can be used in agriculture to increase global food production and enhance food quality. They are suitable for application in different areas of agriculture—such as plant protection, plant growth, stress tolerance, smart delivery systems, crop improvement, biosensors, and precision farming—and in this part of the review, the application of biopolymeric nanomaterials in crop science is highlighted.

#### 3.5.1. Biopolymeric NPs in Plant Growth and Productivity

**Figure 10:**
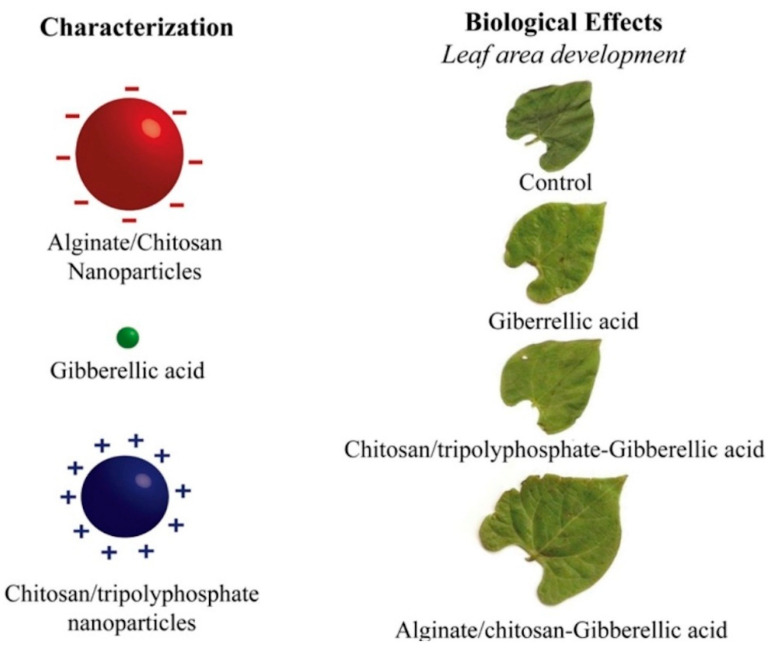
ALG/CS and CS/TPP nanoparticles, gibberellic acid, and the effects of treatment with GA, ALG/CS–GA, and CS/TPP–GA nanoparticles on the leaves’ area. Reproduced with permission from [140], 2017, Elsevier.

One of the main reasons for low crop productivity is environmental factors, such as low temperature, pests, and weeds. Thus, it is important to find growth promotors and biosensors that can be used to effectively assess the biochemical and physiological changes in plant growth. Nanomaterials, including biopolymeric nanoparticles, can be used as delivery systems for plant growth promoters to increase their efficacy. In their work, Santo Pereira, A.E. et al. developed two systems composed of alginate/chitosan (ALG/CS) or chitosan/tripolyphosphate (CS/TPP) and used them to deliver the plant hormone gibberellic acid (GA). These systems showed effects on both morphological and biochemical parameters [140]. At the biochemical level, chitosan nanoparticle systems had different effects on the pigment contents, as the ALG/CS–GA nanoparticles increased the levels of chlorophyll “a”, relative to the control, while the CS/TPP–GA nanoparticles increased the levels of chlorophyll “b”. Regarding the content of carotenoids, only the ALG/CS–GA nanoparticles were found to increase it [140]. Morphological parameters, such as leaf area and root length, were also important. Roots are critical for plant growth, as they are responsible for the uptake of water and nutrients [141,142,143,144], and the CS/TPP–GA nanoparticles were found to increase the root length by 5.5 and 5.5 cm at concentrations of 0.012 and 0.025%, respectively, compared to the control. The free hormone showed an increase in root length of 4 cm at concentrations of 0.025% [140]. Regarding the leaf area, a large leaf area increases the photosynthetic activity of the plant and, therefore, results in an increase in the energy available for plant development. The experiment showed that the use of ALG/CS–GA nanoparticles at concentrations of 0.012 led to greater leaf area compared to other variants (Figure 10) [140]. 

The added advantage of the use of such chitosan-based nanosystems is that studies have shown improved antifungal activity of chitosan nanoparticles [145,146,147]; thus, they can help improve the plant growth while also protecting the crop. 

Apart from the delivery of plant growth promoters, biopolymeric nanoparticles have also been shown to have growth-promoting properties. Several studies have shown that chitosan nanoparticles promote seed germination in crops and can therefore be used to produce healthy seedlings [148,149,150]. 

Kadam et al. also showed the effect of chitosan-based nanoparticles on plant growth by developing salicylic acid–chitosan nanoparticles (SA–CS NPs). Their application increased the activities of seed reserve food-remobilizing enzymes during seedling growth, such as α-amylase and proteases, with higher enzyme activities observed from the 3rd to the 7th days. In addition, they also observed that the shoot–root length and fresh weight were significantly higher in nanoparticle treatments, compared to the control, bulk chitosan, and salicylic acid treatments [151]. 

Chitosan nanoparticles also effect the germination and seedling growth of wheat (*Triticum aestivum* L.), as shown by the work of Li et al., who showed that the chitosan nanoparticles had a positive impact on the seed germination and seedling growth of wheat at a lower concentration compared to chitosan treatment. Such efficacy was suggested to be due to the high adsorption of chitosan nanoparticles on the surface of wheat seeds observed via energy-dispersive spectroscopy and confocal laser scanning microscopy [149].

In addition to laboratory experiments on seedling growth, studies in greenhouses also showed the effects of chitosan nanoparticles, as their application increased the chlorophyll content and the photosynthesis intensity in coffee leaves in comparison to controls. They also improved the nutrient uptake and had a significant impact on the growth of the coffee seedlings, with the growth parameters of the treated coffee higher than those of the controls [152].

In addition to the treatment of seeds, foliar application of biopolymeric nanoparticles improves plant growth. Chitosan nanoparticles loaded with macronutrients—nitrogen, phosphorus, and potassium (NPK)—were easily applied to the leaf surfaces of wheat plants, and they entered the stomata via gas uptake. Transmission electron microscopy showed that the nanoparticles were taken up and transported through phloem tissues. The foliar application of the nanoparticles with NPK increased the wheat yield [153]. 

Loading or encapsulating nutrients in biopolymeric nanoparticles can also help minimize economical losses and control the release of nutrients. In addition to chitosan, other carbohydrate- and protein-based polymeric nanoparticles have been used in the preparation of such systems for the controlled release of nutrients [154,155,156,157]. 

Overall, biopolymeric nanoparticles can be used to promote plant growth and productivity, but their mode of application and efficacy may vary depending on the type of plant species and their growth stages.

#### 3.5.2. Biopolymeric NPs in Plants’ Defense against Pathogens and Abiotic Stress Factors

Nanoparticles in plant protection can be used either for the delivery of pesticides or for the rapid diagnosis of plant diseases [158], and the ecological safety of bio-based nanomaterials makes them more attractive for use in plant protection. In addition, several studies have shown that the use of biopolymeric nanomaterials upregulates defense enzymes/genes in plants and protects crops from pathogens [139,159,160]. Among them, chitosan and other carbohydrate-based nanoparticles have shown their efficacy in crop protection [154,161]. 

Among them, β-d-glucan nanoparticles showed antifungal activity against *P. aphanidermatum*—a devastating fungus that affects major crop plants [162,163,164]. Chitosan nanoparticles have also been found to be able to suppress blast disease of rice, caused by the fungus *Pyricularia grisea* [165]. 

The antifungal activity of chitosan nanoparticles was studied against rice sheath blight disease caused by the pathogenic fungus *Rhizoctonia solani*, and it was determined that the use of chitosan nanoparticles led to an increase in the levels of defense enzymes, such as peroxidase, phenylalanine ammonia-lyase, and chitinase enzymes, thus showing that these are potent plant immunity boosters that are able to help suppress up to 90% of disease in detached leaf assay and 75% under greenhouse conditions [166]. 

In their studies, Choudhary et al. showed that chitosan nanoparticles can inhibit the plant pathogen *Fusarium solani* under in vitro conditions [167], while Chen et al. showed that the antimicrobial activity of chitosan-based nanoparticles is due to their zeta potential, which plays a key role in binding with negatively charged microbial membranes [168].

In addition to chitosan nanoparticles, nanocomposites of other biopolymers have also shown antimicrobial properties. Pinto et al. showed the antifungal activity of nanocomposite thin films of pullulan and silver against *Aspergillus niger* [169]. 

Biopolymeric nanoparticles have also been used against plant nematodes, such as the pine wood nematode, which is devastating to pine trees. In their study, Liang et al. tried to improve the poor availability of the widely used bionematicide avermectin—due to its poor solubility in water and rapid photolysis—by encapsulating it within nanoparticles composed of poly-γ-glutamic acid and chitosan. Their results showed that the encapsulation of avermectin within biopolymeric nanoparticles reduced its losses through photolysis by more than 20.0%, and that the mortality rate of nematodes that were treated with 1 ppm of avermectin encapsulated in nanoparticles was 98.6% [170]. 

In addition to the direct antifungal activity of polymeric nanoparticles, they can also be used to control pathogens by controlling the fungicides, as shown by the work of Liu et al., who used nanoparticle systems based on the polymeric materials polyvinylpyridine and polyvinyl pyridine-co-styrene to slowly release the fungicides tebuconazole and chlorothalonil to control a common brown rot wood decay fungus [171]. Other than these polymers and inorganic nanoparticles used as delivery systems for agrochemicals [172], several reports have highlighted the preparation and application of chitosan-based nanoparticles for the delivery and controlled release of pesticides in agriculture [137]. In these studies, chitosan-based nanomaterials have been used for the delivery of insecticides such as rotenone [173] and imidacloprid [174], the biopesticide azadirachtin [175], and the essential oil of *Lippia sidoides*, with insecticidal properties [176].

Another interesting use of biopolymeric nanoparticles is in the controlled release of herbicides, since they can increase herbicide efficacy at reduced doses, thus decreasing the environmental impact of said herbicides. Several studies have shown the efficacy of chitosan-based nanomaterials in the delivery of herbicides. Silva et al. used alginate/chitosan nanoparticles for the delivery of paraquat herbicides [177], while Grillo et al. used chitosan/tripolyphosphate nanoparticles as carriers of the same herbicide [137,178]. The results of these studies showed that biopolymeric nanoparticles were able to decrease the herbicides’ toxicity, thus reducing the negative impacts caused by paraquat [137]. 

Many of these studies show that biopolymer-based nanotechnologies should be considered as useful tools for environmental protection against the negative impacts of agrochemicals.

**Figure 11:**
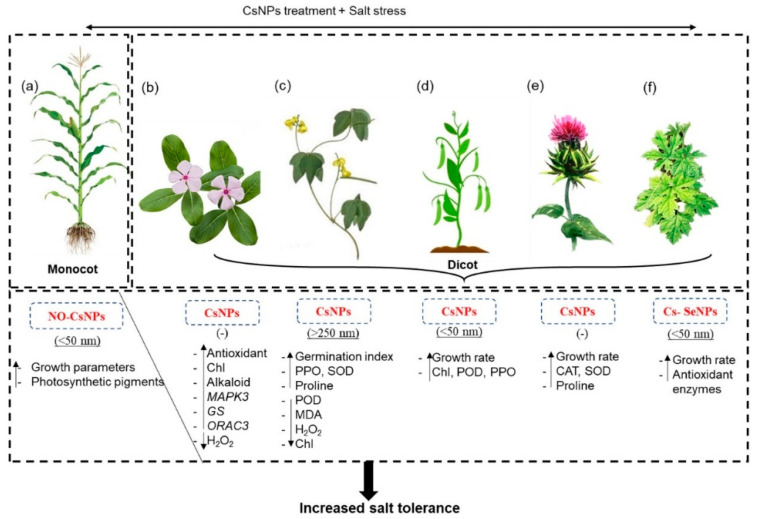
Treatment with CsNPs/modified CsBMs under salinity stress improved the plant performance in various plant species (**a**) maize, (**b**) periwinkle, (**c**) mung bean, (**d**) bean, (**e**) milk thistle, (**f**) bitter melon. Reproduced with permission from [182], 2022, Elsevier.

Along with plant pathogens, abiotic stress factors—such as salinity and drought—are another reason for low crop productivity. Chitosan nanoparticles have been reported to improve the salinity stress response in several plants [179,180,181]. In their work, Balusamy et al. discussed the beneficial effect of chitosans, chitosan nanoparticles (CsNPs), and modified chitosan biomaterials (CsBMs) under salt stress to improve crop performances by stimulating physiological changes in plants, including changes in primary metabolite production, the jasmonic acid signaling pathway, antioxidant activity, secondary metabolite production, and membrane permeability, leading to increased salt resistance (Figure 11) [182].

In addition to salt tolerance, chitosan nanoparticles have also shown beneficial effects for plants under drought conditions [183]. The results of the work of Behboudi et al. indicated that the application of chitosan NPs—especially at 90 ppm—can mitigate adverse effects of drought on wheat [184]. Ali et al. reported that the application of chitosan NPs mitigated the negative effects of drought stress, such as the reduction in relative water content, stomatal conductance, and total chlorophyll. NPs increased the activity of the enzymes catalase and ascorbate peroxidase, as well as the accumulation of proline. They also reduced the oxidative stress and the accumulation of H_2_O_2_ and malondialdehyde, thus preserving membrane integrity. Their results also showed an increase in the accumulation of alkaloids, which was associated with the expression of genes of the alkaloid biosynthetic pathway—strictosidine synthase, deacetylvindoline-4-O-acetyltransferase, peroxidase 1, and geissoschizine synthase [185]. 

From those works, we can see that chitosan nanoparticles help mitigate the negative effects of abiotic stress through biochemical changes and gene expression modulation.

#### 3.5.3. Biopolymeric NPs in Crop Improvement

Crop improvement via genetic engineering and genomic editing can help speed up the development of improved crops. One of the challenges in plant engineering is gene delivery through plant cell walls. The traditional gene transfer methods—such as Agrobacterium-mediated gene transfer, electroporation, PEG-mediated gene transfer, particle gun bombardment, and others—are expensive [186], and can cause significant perturbation to the growth of cells [137]. Recent knowledge and technology have enabled the use of genome editing in crop improvement, making it simple and precise by increasing the precision of the correction or insertion, and preventing cell toxicity [187]. 

Several studies have reported the use of nanomaterials in genetic engineering and genome editing [188,189,190,191,192,193]. Nanomaterials have enabled efficient, targeted delivery into diverse plant species and tissues—both monocot and dicot plants—contributed to the controlled release of DNA and proteins and their protection from degradation, and in some cases even allowed imaging of the delivery and release processes in plants [193,194].

Biopolymeric nanoparticles, such as chitosan nanoparticles, have been used as nanocarriers for the delivery of DNA, RNA, and proteins in genetic engineering and the CRISPR (clustered regularly interspaced short palindromic repeats)/Cas9 (CRISPR-associated protein 9) system for genome editing [195,196]. Duceppe and Tabrizian showed the potential of chitosan NPs for DNA delivery, since they can effectively condense and complex DNA through the electrostatic interactions between the positively charged amino groups on the glucosamine units of chitosan and the negatively charged phosphates on DNA [197]. In addition, it was reported that chitosan-based delivery systems can protect DNA from nuclease degradation [198].

Kwak et al. used chitosan-complexed single-walled nanotubes for the transformation of chloroplasts. They demonstrated the potential of nanoparticle-mediated chloroplast transgene delivery tools, which can be used to transform mature plants. Their chitosan-nanoparticle-mediated chloroplast transgene delivery platform was noted as simple, easy to use, cost-effective, and applicable to mature plants across different species, and did not require specialized, expensive equipment, enabling its widespread application in plant bioengineering and plant biology studies [199]. The authors also hypothesized that their approach could be used in genome editing for the delivery of zinc-finger nucleases, transcription activator-like effector nucleases (TALENs), and clustered regularly interspaced short palindromic repeats (CRISPR)–CRISPR-associated protein 9 (Cas9) vectors. 

**Figure 12:**
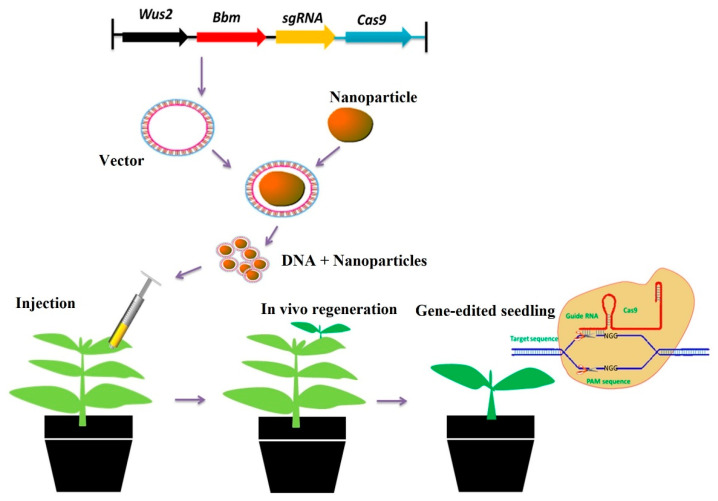
Illustration of prospective applications of nanoparticle-based delivery of CRISPR/Cas9 in plant engineering. Reproduced with permission from [191], 2020, John Wiley and Sons.

Since several studies have suggested that the *Bbm* and *Wus2* genes can be used to improve the efficiency of plant engineering and promote plant regeneration [191,200,201], Lv et al. suggested that nanoparticles can be used to carry these genes—*Wus2* and *Bbm*—and CRISPR/Cas9 into intact plant cells, thus producing a transgenic plant without the plant tissue culture processes (Figure 12) [191].

Overall, recent studies show the potential advantages of using nanoparticles, including biopolymeric nanoparticles, to deliver biomolecules (e.g., DNA/RNA/proteins/ribonucleoproteins) in plants to accelerate the process of genetic transformation and the development of improved plant genotypes. They can even help in the transformation of crops that are non-compatible with traditional transformation methods and tissue culture protocols. In addition to the applied research, biopolymeric nanoparticles can also help advance the fundamental understanding of plants’ cellular and organellar genetics, and aid in the discovery of new genes and gene clusters, as well as epigenetic indicators of plant metabolism.

## 4. Conclusions

Nanoparticles have been developed from various materials, such as synthetic polymers and natural biological polymers. Biopolymeric nanoparticles offer several advantages, such as biodegradability, biocompatibility, and reduced toxicity. This review presents the main methods for the preparation of biopolymeric nanoparticles, such as ionic gelation, nanoprecipitation and microemulsion methods, spray-drying, etc. The promising applications of biopolymeric nanoparticles are also presented, with a special emphasis placed on biomedical and agricultural applications. For the biomedical applications, we present the potential use of biopolymeric NPs in drug delivery and diagnostic systems, as well as in tissue engineering, and photodynamic and photothermal therapy. Moreover, we also highlight other advantages, as well as the possibility of creating antibacterial systems based on biopolymeric nanoparticles against many microbial strains. The potential use of biopolymeric nanoparticles in agriculture is also presented, with several studies reporting on the growth-promoting properties of several NPs. In addition, biopolymeric NPs can also be used as delivery systems for agrochemicals, and in crop improvement.

The cytotoxicity, biodegradability, and biocompatibility of biopolymeric nanoparticles enable their use in various fields, and the present review was designed to present recent advances that could help researchers in the large-scale production of these nanoparticles and—by combining sciences such as bioengineering, chemical modification, and nanotechnology—aid in the development of nanoparticles with improved efficiency and effectiveness, thus increasing their applicability.

## Data Availability

The data presented in this study are available in the article.
